# Efficacy of Concurrent Chemotherapy for Intermediate Risk NPC in the Intensity-Modulated Radiotherapy Era: a Propensity-Matched Analysis

**DOI:** 10.1038/srep17378

**Published:** 2015-11-27

**Authors:** Fan Zhang, Yuan Zhang, Wen-Fei Li, Xu Liu, Rui Guo, Ying Sun, Ai-Hua Lin, Lei Chen, Jun Ma

**Affiliations:** 1 State Key Laboratory of Oncology in South China, Collaborative Innovation Center for Cancer Medicine, Department of Radiation Oncology, Sun Yat-sen University Cancer Center, People’s Republic of China; 2 Department of Radiation Oncology, The Sixth Affiliated Hospital of Sun Yat-sen University, People’s Republic of China; 3 Department of Medical Statistics and Epidemiology, School of Public Health, Sun Yat-sen University, No.74 Zhongshan Road, Guangzhou, People’s Republic of China

## Abstract

This study is to evaluate the efficacy of additional concurrent chemotherapy for intermediate risk (stage II and T3N0M0) NPC patients treated with intensity-modulated radiotherapy (IMRT).440 patients with intermediate risk NPC were studied retrospectively, including 128 patients treated with IMRT alone [radiotherapy group (RT group)] and 312 paitents treated with IMRT plus concurrent chemotherapy [chemoradiotherapy group (CRT group)]. Propensity score matching was carried out to create RT and CRT cohorts equally matched for host and tumor factor. Significantly more severe acute toxicities were observed in the CRT group than in the RT group. Multivariate analyses of 440 patients failed to demonstrate concurrent chemotherapy as an independent prognostic factor for FFS, LR-FFS, and D-FFS. Between the well-matched RT cohort and the CRT cohort, no significant difference was demonstrated in all survival endpoints (FFS: 92.8% versus 91.2%, *P* = 0.801; LR-FFS: 95.2% versus 94.4%, *P* = 0.755; D-FFS: 96.4% versus 96.3%, *P* = 0.803; OS: 98.2% versus 98.9%, *P* = 0.276). Our results demonstrated that for patients with intermediate risk NPC treated with IMRT, additional concurrent chemotherapy did not provide any significant survival benefit but significantly more severe acute toxicities. However, prospective randomized trials are warranted for the ultimate confirm of our findings.

Nasopharyngeal carcinoma (NPC) is an endemic disease in south China and a highly chemoradiosensitive tumor. RT is the primary modality of treatment for nondisseminated NPC. RT alone can achieve excellent survival in early stage (stage I) patients, while the survival of patients with stage II NPC remains relatively unsatisfactory^1^,^2^. Currently, concurrent chemoradiotherapy with/without sequential chemotherapy (i.e., induction or adjuvant chemotherapy) is the standard treatment modality for stage II NPC according to the National Comprehensive Cancer Network (NCCN) guideline. There has been one randomized trial^3^ performed in the stage II population that demonstrated improvement in distant control and overall survival (OS) after the addition of concurrent chemotherapy. While retrospective studies showed no benefit in all endpoints^4^ or benefit in distant control and OS^5^ from induction chemotherapy, or only improved locoregional control from concurrent chemotherapy^6^. Remarkably, all these studies were based on two-dimensional conventional radiotherapy (2DCRT).

As one of the key milestones in the management of NPC, intensity-modulated radiotherapy (IMRT) offers improved tumor target conformity, higher dose to the target, superior radiobiological effect of accelerated fractionation, and better protection of normal organ at risk^7^,^8^; therefore it has gradually replaced 2DCRT and changed the treatment modality of NPC. With better treatment outcomes from IMRT than 2DCRT^9^,^10^,^11^, the differential gain in survival from additional chemotherapy was speculated to be smaller within the framework of IMRT^12^,^13^. A previous study has showed us inspiring long-term survival of stage II patients with IMRT alone, exceeding 90% in all endpoints^14^. However, for the only two studies that investigated the efficacy of additional chemotherapy for this population treated with IMRT, the results were conflicting and the study samples were small^13^,^15^. Thus, the sparse available evidence addressed this issue was debatable and of limmited value for clinical reference. Additionally, the addition of platinum-based chemotherapy obviously increased severe adverse-effects^3^,^6^,^12^,^16^,^17^, the risk of treatment-related mortality^18^, and costs. Therefore, the possibility to omit chemotherapy in this subgroup of patients was appealing in case of the absence of survival benefit. Moreover, better local control of IMRT has also changed the hazard distribution for prognoses of NPC^19^. For example, the stage T3N0M0 subgroup has been reported to have similar survival to stage II in the modern era^19^,^20^. Given that, we included stage II and T3N0M0 disease as intermediate risk NPC in the era of IMRT in our study.

Therefore, our team conducted a large-sample retrospective study to evaluate the efficacy of additional concurrent chemotherapy for intermediate risk NPC treated with IMRT in our center in an endemic area.

## Material and Methods

### Patient Selection

1,811 consecutive patients with newly diagnosed nonmetastatic NPC treated with IMRT at the Sun Yat-sen University Cancer Center (Guangzhou, People’s Republic of China) between November 2009 and December 2012 were studied retrospectively. All clinical records and magnetic resonance imaging (MRI) materials were reviewed by two radiologists with more than 10 years of experience in head and neck cancers. All scans were evaluated independently and disagreements were resolved by consensus. All patients were re-staged according to the 7th edition of the American Joint Committee on Cancer (AJCC) Staging System for NPC^21^. Of these, 486 patients were restaged as stage II and T3N0M0. Fourty-six (9.5%) patients who received induction or adjuvant chemotherapy alone without concurrent chemotherapy were subsequently eliminated from the study. The resulting 440 patients were incorporated in the study, including 128 in the RT group and 312 in the chemoradiotherapy (CRT) group. This retrospective study was approved by the Institutional Review Board of Sun Yat-sen University Cancer Center and in accord with the institutional policy to protect the patients’ private information. The need for informed consent was waived.

### Radiotherapy

IMRT treatment details have been previously reported^9^. Target volumes were delineated according to our institutional treatment protocol^9^, which is in agreement with the International Commission on Radiation Units and Measurements Reports 50 and 62. The clinical target volumes (CTV) were individually delineated based on the tumor invasion pattern. The prescribed radiation dose was defined as follows: a total dose of 66–72 Gy to the planning target volume (PTV) of the gross tumor volume of the primary (GTV-P), 64–70 Gy to the PTV of the nodal gross tumor volume (GTV-N), 60–63 Gy to the PTV of CTV-1 (i.e., high risk regions), 54–56 Gy to PTV of CTV-2 (i.e., low-risk regions) and CTV-N (i.e., neck nodal regions). All patients were treated with one fraction daily over 5 days per week.

### Toxicity and follow-up

Acute and late toxicities were documented according to the Common Terminology Criteria for Adverse Events version 3.0 and/or the Radiation Morbidity Scoring Criteria of the Radiation Therapy Oncology Group. The duration of patient follow-up was measured from the first date of treatment to either the date of death or the date of last examination. Patients were examined and followed-up at least every 3 months during the first 2 years, and thereafter every 5 months for up to 3 years or until death.

### Statistical analysis

Our primary endpoint was failure-free survival (FFS). Our secondary endpoints were OS, locoregional failure-free survival (LR-FFS), and distant failure-free survival (D-FFS). FFS was calculated from the first date of treatment to the date of treatment failure or death from any cause, whichever occurred first; OS, to last examination or death; and LR–FFS and D–FFS, to first locoregional or remote failure, respectively.

The chi-square test (or Fisher’s exact test, if indicated) was used to test the baseline balance and toxic effect rates over two groups and/or cohorts. The estimated survival rates were calculated using the Kaplan–Meier method and differences were compared using the log-rank test. Multivariate analyses using the Cox proportional hazard model were used to test independent significance using backward elimination of insignificant explanatory variables. Covariates included host factors (i.e., sex, age), tumor factors (i.e., T and N classification), and chemotherapy intervention (i.e., CRT group).

Since patient selection bias might be one of the explanations for the equally excellent survival results in both groups, propensity score (PS) matching, an effective technique for adjusting bias, was used create two cohorts equally matched for host and tumor factors^22^. The PS was developed using sex, age, T stage, N stage, GTV-P level, and pretreatment plasma Epstein–Barr Virus DNA (pEBV DNA) level^23^,^24^. We carried out a two-to-one propensity matching using the caliper match algorithm, with sampling without replacement and caliper width set to 0.2 to yield sufficient power and similarity between the CRT and RT cohorts^22^,^25^.

The criterion for statistical significance was set at α = 0.05. *P*-values were determined from two-sided tests. All analyses were carried out with the Stata Statistical Computer Package (STATA 10; StataCorp LP, College Station, Texas, USA).

## Results

### Patient and chemotherapy characteristics

Table 1 shows the not perfectly well balanced baseline characteristics between the two groups, as well as chemotherapy regimen in the CRT group. With respect to concurrent chemotherapy, 172/312 patients (55.4%) received a 3-weekly platinum-based regimen and 94.8% of them received at least two cycles of chemotherapy. Furthermore, 126/312 (39.4%) received a weekly platinum-based regimen and 72.2% of them received at least five cycles, respectively. Further chemotherapy details were available in Table 2

### Survival outcome

The median follow-up was 37.3 months (range 8.0–58.8 months). 432 participants (98.2%) were followed up for more than 2 years. We observed 49 events and 46 treatment failures. A summary of failure patterns is displayed in Table 3.

No statistically significant difference was observed in the estimated 3-year FFS, LR-FFS, D-FFS, and OS rates between the RT group and the CRT group (FFS: 92.9% versus 86.7%, *P* = 0.140; LR-FFS: 95.0% versus 92.6%, *P* = 0.374; D-FFS: 96.9% versus 93.0%, *P* = 0.128; OS: 98.4% versus 97.7%, *P* = 0.910, Table 3). Additionally, we performed subgroup analyses according to TNM classification (T2N0M0, T1N1M0, T2N1M0, and T3N0M0). We found that additional concurrent chemotherapy failed to result in significant differences in the four subgroups for all endpoints examined (Table 4). For those with pretreatment pEBV DNA level ≥4000 copy/ml, our analyses failed to examed out any survival benefit from concurrent chemotherapy.

CRT group was not an independent prognostic factor for FFS, LR-FFS, or D-FFS using multivariate analyses. However, we did observe that pretreatment pEBV DNA levels ≥4000 copy/ml was an independent prognostic factor FFS and D-FFS; Detectable post-treatment pEBV DNA was also an independent prognostic factor FFS, LR-FFS and D-FFS (Table 5).

### Toxicity

No treatment-related death was observed in our study. Patients in the CRT group experienced significantly higher rate of severe acute toxicities (grade 3–4) than the RT group during RT (42.3% versus 21.1%, *P* < 0.001) and this difference was mainly attributed to mucositis, leucopenia, neutropenia, and gastrointestinal reactions (Table 6). In addition, we noted that more patients in the CRT group had over 5% weight loss (grade 1–4) compared with the RT group (57.4% versus 22.7%, *P* < 0.001). No significant difference in severe late toxicities was observed between the two groups [CRT group (3.2%) versus RT group (2.3%), *P* = 0.628, Table 6].

### Role of concurrent chemotherapy

**Figure 1:**
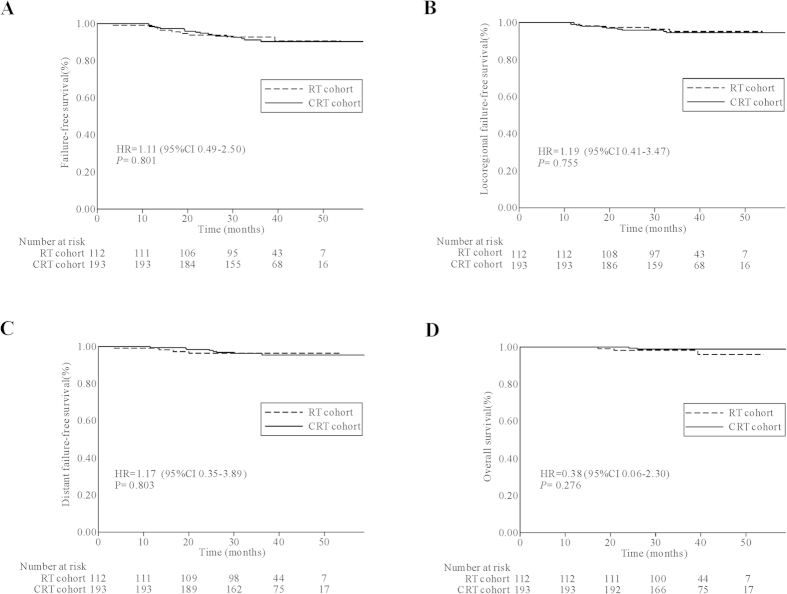
Kaplan–Meier survival curves for the matched RT and CRT cohorts. Failure–free survival (**A**), locoregional failure–free survival (**B**), distant failure–free survival (**C**), and overall survival (**D**). Hazard ratios (HRs) were calculated with the unadjusted Cox proportional hazards model; *P*-values were calculated by the unadjusted log–rank test. RT = radiotherapy; CRT = chemoradiotherapy.

As shown in Table 1 and Table 3, with more patients with N1 disease than the RT group, the CRT group experienced slightly lower, though not statistically significant, survival than the RT group despite the additional concurrent chemotherapy these patients received. We then carried out PS matching and the resulting two well-balanced cohorts were shown in Table 1. As displayed in Fig. 1, no difference was observed in the estimated 3-year FFS, LR-FFS, D-FFS, or OS between the RT cohort and the CRT cohort (FFS: 92.8% versus 91.2%, *P* = 0.801, Fig. 1A; LR-FFS: 95.2% versus 94.4%, *P* = 0.755, Fig. 1B; D-FFS: 96.4% versus 96.3%, *P* = 0.803, Fig. 1C; OS: 98.2% versus 98.9%, *P* = 0.276, Fig. 1D).

## Discussion

To our knowledge this is the first large-sample comparison study between IMRT alone and IMRT plus concurrent chemotherapy in intermediate risk NPC. By conducting multivariate analyses and PS matching to adjust the bias, our data still fail to prove any significant survival improvement from concurrent chemotherapy in addition to IMRT in all endpoints. Moreover, significantly more severe acute toxicities were observed in the CRT group.

There are three possible explanations for our negative findings. Firstly, the stronger benefit of concurrent chemotherapy in locoregional control and overall survival by enhancing the local effect of radiotherapy has been proven and established by numerous trials^12^,^16^,^17^ and meta-analyses^26^,^27^ in the 2DCRT era. However, a substantial improvement in treatment outcomes with IMRT compared with 2DCRT has been shown primarily in LR-FFS^9^,^10^,^11^ in NPC patients. This might have narrowed any potential therapeutic gain in locoregional control offered by concurrent chemotherapy. Additionally, the improved locoregional control from concurrent chemotherapy in 2DCRT era may have a favorable influence on the distant control, which might has been likely substituted by IMRT. Moreover, there have been some studies^4^,^6^,^12^,^13^,^17^ and meta-analysis^26^ that demonstrated the ineffectiveness of concurrent platinum-based chemotherapy for the eradication of micro metastases. The above reasons might contribute together to the absence of improved distant control.

Secondly, it is possible that the extra severe acute hematologic and nonhematologic toxicities from concurrent chemotherapy were harmful to patient’s prognosis. There has been evidence that severe treatment related lymphopenia was associated with poor progression free survival in patients with squamous cell head and neck cancer^28^. Moreover, notably 30% more patients in the CRT group experienced over a 5% weight loss during RT, which was found to be the only independent factor associated with poor survival in a retrospective study of NPC and the possible reason might be the unfavorable impact of weight loss on treatment including compliance^29^,^30^ and less accuracy in patient position for IMRT^31^,^32^. Therefore, despite tolerable these toxicities may seem to be, them might have further compromised the therapeutic ratio of concurrent chemotherapy in this population.

Finally, as the main criteria for prediction patient’s prognosis, the present NPC AJCC staging system has been widely used in clinic practice for reseanable treatment stratification and in clinical trials for target patient selection, as well as in our study. We should aware that the AJCC staging system is restricted in its diagnostic reach to the anatomical extent of the tumors, and not accurate enough to categorize patients at intermediate risk of disease recurrence. An increasing number of promising indictors have been studied to improve the TMN staging system, such as biological, genetic, and molecular prognosis factors^29^,^30^. Researchers might consider testing combinations of both anatomical and non-anatomical prognosis factors to achieve optimal selection for concurrent chemotherapy in this population in the future. Similarly, the NPC-0502 study (NCT00370890), a promising clinical trial, was designed to use pEBV DNA level as a selection for chemotherapy regimen.

Interestingly, both pretreatment pEBV DNA level ≥4000 copy/ml and detectable pEBV DNA after treatment are the independent prognostic factor for FFS, D-FFS. These results were cosistent with previos studies^24^,^33^,^34^. However, for those with pretreatment pEBV DNA level ≥4000 copy/ml, our analyses failed to examed out any survival benefit from the current chemotherapy. We prefer to attribute this negective finding to the unadjustable bias and the small sample size of our data: the overwhelming majority of this cohort (89/107, 83.2%) received chemotherapy. We expect the benefit of chemotherapy for this special cohort may be proved in the retrospective or prospective study series with adjustable bias or no bias in the furture. Moreover, detectable post-treatment pEBV DNA was found to be a more strong prognostic factor than pretreatment pEBV DNA level ≥4000 copy/ml in our study, which may be very meaningful for selecting patients at high risk of disease recurrence after radiotherapy for more aggressive treatment. Further investigation was warrant for best treatment for patients with detectable pEBV DNA after treatment.

There were two small-sample retrospective studies performed in the IMRT era. Luo *et al*.^15^ focused on 69 patients with stage I-II NPC and demonstrated an improvement of survival in all endpoints from additional concurrent chemotherapy. Notably, although patients with stage I was included, the locoregional and distant control rate for the patients with IMRT alone remained 81.4–84.0%, far lower than that reported in a previous large-sample study^14^ and in our study. The main reason for this difference may be that *(1)* Luo and colleagues’ study was from a non-endemic area of China, *(2)* 71% of patients involved were with World Health Organization (WHO) II histology and *(3)* the study sample was small. Thus, it should be cautious to apply their findings to endemic area with predominantly WHO III histology, which was found to confer better prognosis^35^. On the contrary, Tham *et al*.^13^ reported no significant improvement in all survival endpoints from chemotherapy of any schedule in 107 patients with stage II NPC. However, they did not focus on concurrent chemotherapy because most patients were treated with induction chemotherapy alone and only 8 patients received concurrent chemotherapy^13^, which is proved to be the most effective chemotherapy regimen to NPC^26^,^27^ and most widely-used in clinical practice to attempt better survival according to the influential NCCN guideline. Therefore, their findings may not be representative evidence for the efficacy of CRT and of limited persuasion for treatment reconsideration from oncologists.

Oncologists should notice that current NCCN guideline recommendations of chemotherapy for intermediate risk NPC is based on evidence from studies in the 2DCRT era. However our data demostrated that the additional concurrent chemotherapy did not provide any further survival benefit to intermediate risk NPC patients within the framwork of IMRT. Therefore our results might provide important information in clinical decision-making, avoiding overtreatment as well as unnecessary toxicities and costs without hazarding patients’ survival. Our study has some limitations. This is a retrospective study with inevitable selection bias. However, we managed to use PS matching to adjust the bias effectively and the final matched cohort was well balanced on the both host and tumor factors, including known important prognostic factors such as GTV-P level and pretreatment EBV DNA level. Therefore our results should be of credible references to clinical practise and future confirm of optimal treatment modality for intermediate risk NPC. However, it should be awared that PS matching was limitted to unknown prognostic factors; some factors such as economic status, living conditions may affect patients’ treatment choice. However, the collection of these factors was not in the protocal of our centre, which may be the potential hidden bias though PS matching was used in this series. Therefore, we are looking forward to trials to confirm our findings and two prospective phase II randomized trials are underway (NCT00817258 and NCT01187238).

## Conclusion

Our results demonstrated that for patients with intermediate risk NPC treated with IMRT, additional concurrent chemotherapy did not provide any significant survival benefit but significantly more severe acute toxicities, therefore might not be recommended as routine use. However, prospective randomized trials are warranted for the ultimate confirm of our findings.

## Additional Information

**How to cite this article**: Zhang, F. *et al*. Efficacy of Concurrent Chemotherapy for Intermediate Risk NPC in the Intensity-Modulated Radiotherapy Era: a Propensity-Matched Analysis. *Sci. Rep*. **5**, 17378; doi: 10.1038/srep17378 (2015).

## Figures and Tables

**Table 1:** Baseline characteristics and treatment details before and after matching.

Characteristics, *n* (%)	Before matching			After matching				
	RT group *n* = 128	CRT group *n* = 312	*p* value	RT cohort*n = *112	CRT cohort *n = *193	*p* value		
Age			0.095			0.629		
<50y	82 (64.1)	225 (72.1)		73 (65.2)	131 (67.9)			
≥50y	46 (35.9)	87 (27.6)		39 (34.8)	62 (32.1)			
Sex			0.652			0.898		
Male	93 (72.7)	220 (70.5)		82 (73.2)	140 (72.5)			
Female	35 (27.3)	92 (29.5)		30 (26.8)	53 (27.5)			
KPS			0.238			0.305		
100–90	124 (96.9)	308 (98.7)		124 (97.6)	192 (99.5)			
70–80	4 (3.1)	4 (1.3)		3 (2.4)	1 (0.5)			
Histology			0.198			0.417		
WHO type I	1 (0.8)	1 (0.3)		1 (0.9)	0 (0)			
WHO type II	10 (7.9)	13 (4.2)		7 (6.2)	10 (5.2)			
WHO type III	117 (91.4)	298 (95.5)		104 (92.9)	183 (94.8)			
Staging^a^			0.060			0.777		
II	108 (84.4)	238 (76.3)		92 (82.1)	156 (80.8)			
III	20 (15.6)	74 (23.7)		20 (17.9)	37 (19.2)			
T classification^a^			0.108		0.900			
T1	45 (35.2)	112 (35.9)		45 (40.2)	80 (41.5)			
T2	63 (49.2)	126 (40.4)		47 (42.0)	76 (39.4)			
T3	20 (15.6)	74 (23.7)		20 (17.9)	37 (19.2)			
N classification^a^			0.011			0.355		
N0	56 (43.8)	97 (31.1)		40 (35.7)	59 (30.6)			
N1	72 (56.2)	215 (68.9)		72 (64.3)	134 (69.4)			
GTV-P level			<0.001			0.768		
Stage II, <19 ml	82 (64.1)	142 (45.5)		66 (58.9)	114 (59.1)			
Stage II, ≥19 ml	26 (20.3)	96 (30.8)		26 (23.2)	42 (21.8)			
Stage III, <19 ml	12 (9.4)	21 (6.7)		12 (10.7)	17 (8.8)			
StageIII, ≥19 ml	8 (6.2)	53 (17.0)		8 (7.1)	20 (10.4)			
Pretreatment pEBV DNA level			0.001			0.569		
<4000 copy/ml	110 (85.9)	223 (71.5)		94 (83.9)	157 (81.3)			
≥4000 copy/ml	18 (14.1)	89 (28.5)		18 (16.1)	36 (18.7)			
Combined chemoradiotherapy regimen (*n = *312 before matching; *n = *193 after matching)								
CCRT		213 (68.3)			137 (71.0)			
ICT + CCRT		95 (30.4)			53 (27.5)			
CCRT + ACT		3 (1.0)			2 (1.0)			
ICT + CCRT + ACT		1 (0.3)			1 (0.5)			
Concurrent chemotherapy regimen (*n = *312 before matching; *n = *193 after matching)								
Weekly Cisplatin		110 (35.2)			73 (37.8)			
3-weekly Cisplatin Cisplatin		138 (44.2)			83 (43.0)			
Nedaplatin or Carboplatin		37 (11.8)			21 (10.8)			
Docetaxel		14 (4.5)			8 (4.1)			
Others		13 (4.2)			8 (4.1)			

Abbreviations: WHO, World Health Organization; KPS, Karnofsky scale; RT, radiotherapy; CRT, chemoradiotherapy; NP, nasopharynx; LN, lymph node; GTV-P, gross tumor volume of the primary; pEBV DNA, plasma Epstein–Barr Virus DNA; CCRT, concurrent chemoradiotherapy; ICT, induction chemotherapy; ACT, adjuvant chemotherapy.

^a^Staging, T classification, N classification were based on the 7th edition of the American Joint Commission on Cancer staging system.

**Table 2:** Details of concurrent chemotherapy (*n* = 312).

Concurrent regimen	*n* (%)	Administered dose per cycle (mg/m^2^)	Total Median dose (mg/m^2^)
Weekly regimen (*n* = 140)			
Cisplatin	110 (35.2)	30–50	180
Nedaplatin	14 (4.5)	20–30	150
Carboplatin	2 (0.6)	100	400
Docetaxel	14 (4.5)	15–35	85
3-Weekly regimen (*n* = 172)			
Cisplatin	138 (44.2)	80–100	160
Nedaplatin	21 (6.7)	80–100	160
Others^a^	13 (4.2)	—	—

^a^Others included nedaplatin, 5-Fu regimen in 7 patients; Cisplatin, 5-Fu regimen in 2 patients; docetaxel, cisplatin regimen in 3 patient; docetaxel, nedaplatin in 1 patient.

**Table 3:** Patterns of failure and survival between the RT and CRT groups.

	RT group *n = *128	CRT group *n = *312	HR (95% CI)*	*P*value†
Failure-free survival				
Events	10 (7.8%)	39 (12.5%)	—	—
Rate at 3 years	92.9%	86.7%	1.68 (0.83 –3.36)	0.140
Locoregional Failure-free survival				
Events	6 (4.7%)	21 (6.7%)	—	—
Rate at 3 years	95.0%	92.6%	1.51 (0.61 –3.73)	0.374
Distant Failure-free survival				
Events	4 (3.1%)	21 (6.7%)	—	—
Rate at 3 years	96.9%	93.0%	2.24 (0.77–6.53)	0.128
Overall survival				
Events	3 (2.3%)	8 (2.6%)	—	—
Rate at 3 years	98.4%	97.7%	1.08 (0.29 –4.07)	0.910

Abbreviations: CRT, chemoradiotherapy; RT, radiotherapy; HR, hazards ratio; 95% CI, 95% confidence interval.

^*^Hazard ratios were calculated with the unadjusted Cox proportional hazards model.

^†^*P* values were calculated with the unadjusted log-rank test.

**Table 4:** Comparison of survival in different subgroups according to stage (*n* = 440).

Endpoints (RT group*v*CRT group)	Subgroup Analysis			
	T2N0 (*n = *59)	T1N1 (*n = *157)	T2N1 (*n = *130)	T3N0 (*n = *94)
FFS				
3-year estimated rate*, %	88.9 *v* 86.7	95.6 *v* 87.5	92.6 *v* 83.0	95.0 *v* 91.1
*P* value^†^	0.854	0.160	0.267	0.913
LR-FFS				
3-year estimated rate*, %	94.4 *v* 91.3	95.1 *v* 94.0	100 *v* 94.8	95.0 *v* 95.0
*P* value†	0.675	0.726	0.211	0.962
D-FFS				
3-year estimated rate*, %	94.4 *v* 90.9	97.8 *v* 93.5	96.3 *v* 91.3	100 *v* 95.8
*P* value†	0.631	0.296	0.413	0.356
OS				
3-year estimated rate*, %	94.4 *v* 90.9	100 *v* 100	100 *v* 97.1	100 *v* 97.3
*P* value†	0.645	—	0.328	0.108

Abbreviations: RT, radiotherapy; CRT, chemoradiotherapy; FFS, failure-free survival; LR-FFS, locoregional failure-free survival; D-FFS, distant failure-free survival; OS, overall survival.

^*^The estimated survival rates were calculated using the Kaplan–Meier method.

^†^*P* values were calculated with the unadjusted log-rank test.

**Table 5:** Multivariate analyses of prognostic factors in intermediate risk NPC (*n* = 440)*.

Variable	Hazards ratio (95% CI)	*P*value†
Failure-free survival		
Sex, women *v* men	1.29 (0.68–2.45)	0.44
Age, ≥50 years *v* <50 years	1.14 (0.63–2.07)	0.66
T classification, T2–3 *v* T1	1.32 (0.72–2.43)	0.37
N classification, N1 *v* N0	0.89 (0.42–1.86)	0.75
Pretreatment pEBV DNA level, ≥4000 copy/ml *v* <4000copy/ml	2.22 (1.26–3.94)	<0.01
Post-treatment pEBV DNA, Detectable *v* Undetectable	7.26 (4.13–12.7)	<0.01
GTV-P level, ≥19 ml *v* <19 ml	1.04 (0.57–1.90)	0.89
Treatment group, CRT group *v* RT group	1.40 (0.69–2.83)	0.36
Locoregional failure-free survival		
Sex, women *v* men	2.24 (1.03–4.91)	0.43
Age, ≥50 years *v* <50 years	1.05 (0.46–2.37)	0.91
T classification, T2–3 *v* T1	1.50 (0.66–3.50)	0.32
N classification, N1 *v* N0	1.16 (0.44–3.07)	0.77
Pretreatment pEBV DNA level, ≥4000 copy/ml *v* <4000copy/ml	1.31 (0.58–3.00)	0.52
Post-treatment pEBV DNA, Detectable *v* Undetectable	6.26 (2.89–13.55)	<0.01
GTV-P level, ≥19 ml *v* <19 ml	1.19 (0.53–2.67)	0.68
Treatment group, CRT group *v* RT group	1.46 (0.59–3.62)	0.41
Distant failure-free survival		
Sex, women *v* men	0.49 (0.17–1.45)	0.20
Age, ≥50 years *v* <50 years	0.75 (0.31–1.81)	0.52
T classification, T2–3 *v* T1	1.07 (0.40–2.88)	0.89
N classification, N1 *v* N0	0.67 (0.25–1.77)	0.42
Pretreatment pEBV DNA level, ≥4000 copy/ml *v* <4000 copy/ml	3.88 (1.76–8.57)	0.01
Post-treatment pEBV DNA, Detectable *v* Undetectable	8.48 (3.84–18.73)	<0.01
GTV-P level, ≥19 ml *v* <19 ml	0.84 (0.36–1.95)	0.68
Treatment group, CRT group *v* RT group	1.54 (0.52–4.57)	0.44

Abbreviations: 95% CI, 95% confidence interval; GTV-P, gross tumor volume of the primary; pEBV DNA, plasma Epstein–Barr Virus DNA; RT, radiotherapy; CRT, chemoradiotherapy.

^*^Multivariate analyses were not carried out for OS because the total numbers of failures were too low to obtain a non-overfit model.

^†^*P* values were calculated with an adjusted Cox proportional-hazards model.

**Table 6:** Major severe acute and late toxicities (*n* = 440).

	RT group (*n* = 128)	CRT group (*n* = 312)	*P* value
	Grade 3 or 4	Grade 3 or 4	
Acute toxicity during RT, *n* (%)			
Hematologic	2 (1.6)	46 (14.7)	<0.001
Leukopenia	1 (0.8)	36 (11.5)	<0.001
Granulocytopenia	1 (0.8)	24 (7.7)	0.004
Thrombocytopenia	0 (0)	7 (2.2)	0.113
Anemia	1 (0.8)	2 (0.6)	1.000
Nonhematologic	25 (19.5)	100 (32.1)	0.008
Mucositis	23 (18.0)	90 (28.8)	0.018
Dermatitis	1 (0.8)	4 (1.3)	1.000
Gastrointestinal reactions	0 (0)	15 (4.8)	0.008
Liver dysfunction	1 (0.8)	1 (0.3)	0.498
Renal impairment	0 (0)	0 (0)	—
Wight loss	0 (0)	1 (0.3)	1.000
Total any	27 (21.1)	132 (42.3)	<0.001
Late toxicity, *n* (%)			
Xerostomia	0 (0)	0 (0)	—
Neck tissue damage	0 (0)	0 (0)	—
Deafness or otitis	3 (2.3)	9 (2.9)	1.000
Neuro damage	0 (0)	1 (0.3)*	1.000
Total any	3 (2.3)	10 (3.2)	0.764

Abbreviations: RT, Radiotherapy; CRT, chemoradiotherapy.

^*^One patient who developed a seizure due to radiation-induced brain damage.
