# Corrigendum: Efficacy of Concurrent Chemotherapy for Intermediate Risk NPC in the Intensity-Modulated Radiotherapy Era: a Propensity-Matched Analysis

**DOI:** 10.1038/srep20748

**Published:** 2016-02-08

**Authors:** Fan Zhang, Yuan Zhang, Wen-Fei Li, Xu Liu, Rui Guo, Ying Sun, Ai-Hua Lin, Lei Chen, Jun Ma

Scientific Reports 5: Article number: 1737810.1038/srep17378; published online: 11272015; updated: 02082016.

This Article contains a typographical error in Affiliation 2. The correct affiliation is listed below:

Department of Radiation Oncology, The Fifth Affiliated Hospital of Sun Yat-sen University, People’s Republic of China

